# A role for the midbrain reticular formation in delay-based decision making

**DOI:** 10.3389/fnsys.2024.1481585

**Published:** 2024-12-04

**Authors:** Yong Sang Jo, Gyeong Hee Pyeon, Sheri J. Y. Mizumori

**Affiliations:** ^1^School of Psychology, Korea University, Seoul, Republic of Korea; ^2^Department of Psychology, University of Washington, Seattle, WA, United States

**Keywords:** reticular formation, midbrain area, delay discounting, decision making, delay-based decision making, impulsivity, T-maze

## Abstract

In many real-life situations, decisions involve temporal delays between actions and their outcomes. During these intervals, waiting is an active process that requires maintaining motivation and anticipating future rewards. This study aimed to explore the role of the midbrain reticular formation (MRF) in delay-based decision-making. We recorded neural activity in the MRF while rats performed delay discounting and reward discrimination tasks, choosing between a smaller, sooner reward and a larger, later reward. Our findings reveal that MRF neurons are integral to maintaining motivation during waiting periods by encoding both the anticipated size and the discounted value of delayed rewards. Furthermore, the inactivation of the MRF led to a significant reduction in the rats’ willingness to wait for delayed rewards. These results demonstrate the MRF’s function in balancing the trade-offs between reward magnitude and timing, providing insight into the neural mechanisms that support sustained motivation and decision-making over time.

## Introduction

Delay discounting refers to the cognitive process by which individuals devalue rewards that are delayed in time, favoring immediate, smaller rewards over larger rewards that require waiting. This phenomenon is crucial for understanding decision-making processes, as it involves evaluating trade-offs between the timing and magnitude of rewards. Several interconnected brain regions contribute to this process. Among these, the prefrontal cortex (PFC) plays a central role, with multiple subregions contributing distinct functions. The orbitofrontal cortex (OFC) integrates reward-related information to assess the value of delayed rewards and support adaptive decision-making (Roesch et al., 2006). The medial PFC (mPFC) is critical for cognitive control and working memory, which help sustain goal-directed behavior during delay periods (Narayanan et al., 2006; Euston et al., 2012), and its impairment leads to difficulties in tasks requiring delayed responses (Narayanan et al., 2006). The amygdala contributes by modulating the emotional and motivational aspects of reward valuation (Baxter and Murray, 2002; Murray, 2007). Additionally, the dopamine system, particularly neurons in the ventral tegmental area (VTA), encodes reward prediction errors and adjusts responses based on changes in expected reward value (Roesch et al., 2007; Fiorillo et al., 2008; Kobayashi and Schultz, 2008; Gan et al., 2010). Although these regions have been extensively studied, it is likely that other brain areas also play critical roles in delay discounting, reflecting the complexity of the neural circuits involved in decision-making related to reward processing.

The reticular formation (RF) consists of a variety of functionally distinct yet interconnected nuclei, forming a network that extends rostrally from the medulla through the pons, to the midbrain. The RF has been implicated in coordinating motor activity (Siegel and McGinty, 1977; Fabre et al., 1983; Peterson, 1984), modulating transitions between sleep and wakefulness (Moruzzi and Magoun, 1949; Steriade and McCarley, 1990), and regulating arousal, vigilance, and attention states (Pragay et al., 1978; Kinomura et al., 1996). In particular, the midbrain portion of the RF (MRF) has been suggested to signal elevated motivation in anticipation of positively reinforcing events in rodents (Olds et al., 1969; Phillips and Olds, 1969) and primates (Pragay et al., 1978; Ray et al., 1982). Research has shown that MRF neurons increased firing rates when animals were waiting for anticipated rewards, depending on their motivational states. For example, when animals were food-deprived, MRF neurons exhibited increased activity in anticipation of food but not water. Conversely, in a state of water deprivation, these neurons showed heightened firing rates in anticipation of water but not food. Such neuronal activity suggests that the MRF processes expected rewards and may influence decision-making involving delays. However, the specific role of MRF neurons in delay discounting tasks remains unexplored.

To address this gap, we recorded single-unit activity from the MRF in rats as they performed a delay discounting task, choosing between a smaller, sooner reward (SS) and a larger, later reward (LL). This allowed us to investigate how MRF neurons respond to variations in delay length and reward size. We also used muscimol, a GABA receptor agonist, to inactivate the MRF and observe its effects on the rats’ decision-making behavior. Our findings suggest that MRF neurons encode both the size of upcoming rewards and the discounted value of delayed rewards, playing a crucial role in maintaining motivation during waiting periods.

## Materials and methods

### Subjects

Fifteen male Long–Evans rats (340–400 g; 3–5 months old; Simonson Labs, Gilroy, CA) were housed individually in Plexiglass cages and kept on a 12-h light/dark cycle (lights on at 7:00 AM). Each rat was maintained on a restricted diet at 85% of its free-feeding weight with water freely available. All animal care and experiments were conducted during the light phase, in accordance with the University of Washington’s Institutional Animal Care and Use Committee guidelines.

### Behavioral apparatus

**Figure 1:**
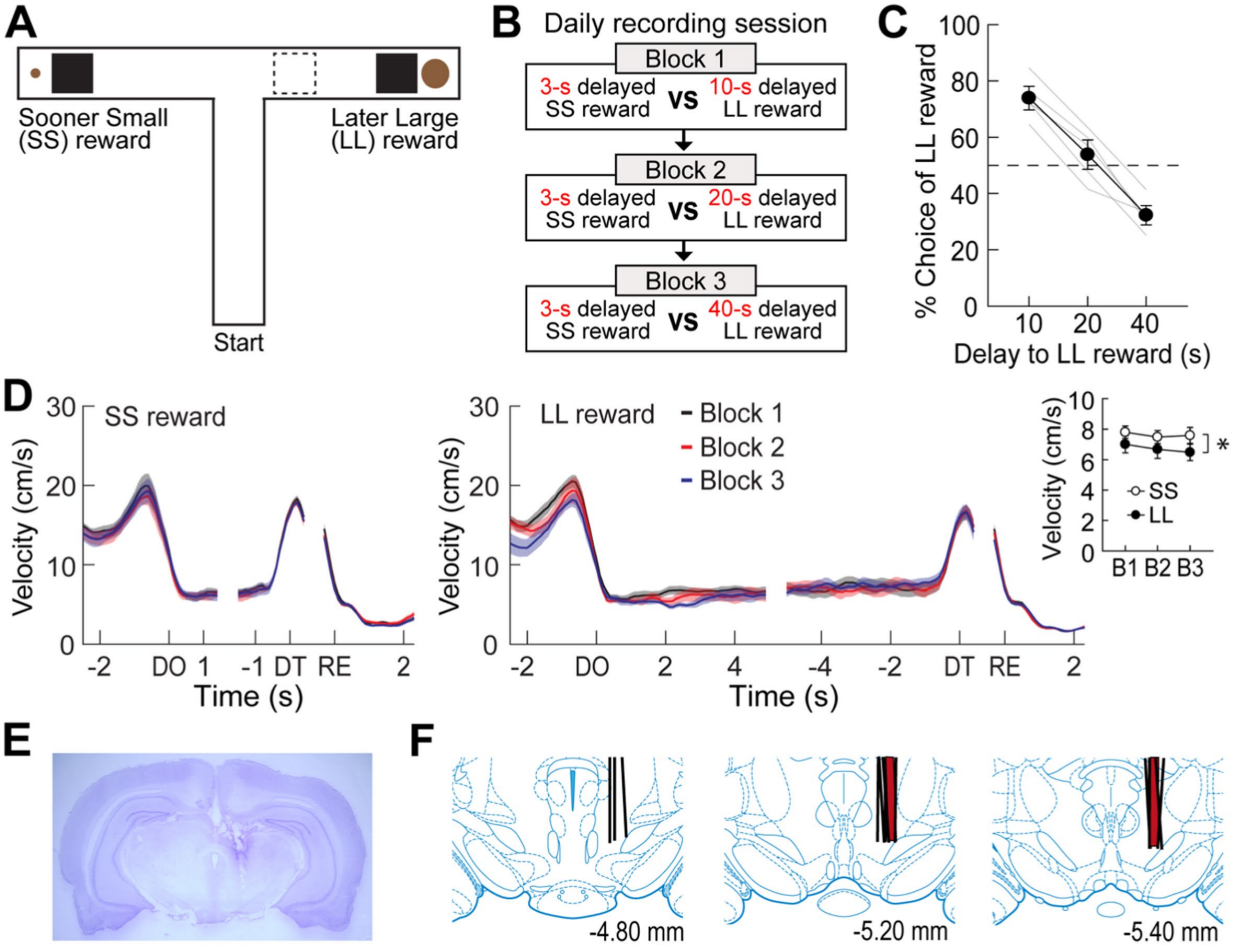
Choice performance on a delay-based decision-making task. **(A)** Illustration of the T-maze. LL and SS rewards were baited at the end of two opposite goal arms. A rectangular wooden barrier (black square) was placed before each food cup to control the animals’ access to rewards. When rats chose a goal arm associated with the LL reward, an additional barrier (indicated by the dashed rectangle) was placed at its entrance to prevent the animals from exiting the goal arm during the delay. **(B)** Daily experimental procedures. Three different lengths of delay to the LL reward were randomly ordered and tested in separate blocks of trials. The delay to the SS reward remained unchanged. Each block consisted of forced-choice trials, followed by free-choice trials. **(C)** Choice preference for the LL reward as a function of the delay to the LL reward. **(D)** Changes in instantaneous velocity around waiting periods. Data are aligned on delay onset (DO), delay termination (DT), and reward (RE). The inset plot shows average velocities during the entire waiting periods. **(E)** Nissl-stained section showing the final location of a tetrode tip in the MRF. **(F)** Reconstruction of all tetrode tracks. MRF neurons in the delay discounting and reward discrimination tasks were recorded from the tetrodes in black and red, respectively. Shaded areas and error bars indicate mean ± SEM.

An elevated T-maze (79 cm from the floor) was used throughout the experiments. The maze, made of black Plexiglass, consisted of one start (the middle stem) and two goal arms (58 × 5.5 cm each), each with a metal food cup at the end (Figure 1A). A black wooden barrier (15 cm width) or an opaque guillotine door was placed before the food cup to control animals’ access to reward during various lengths of waiting periods. Each maze arm was hinged such that its proximal end, closest to the maze center, could be raised and lowered by remote control. The maze was encircled by black curtains that were decorated with spatial cues.

In all experiments, a black barrier was placed 15 cm away from the food cup to prevent the animals from accessing the reward until the designated delay period had passed. This setup introduced a temporal gap, allowing for the distinction between the delay termination and the initiation of the reward response. Once the barrier was removed, the animals could access the reward, which was automatically recorded using a lickometer to ensure precise measurement.

### Presurgical training

All rats were acclimated to the T-maze for 3–5 days. During the habituation phase, they were allowed to freely forage for chocolate milk drops randomly scattered on three maze arms. Then, they were trained to collect a shortly delayed reward (0.15 mL) only from the goal arms. Specifically, each rat was placed on the start arm in a given trial and encouraged to choose one of the goal arms. Upon arrival at the barrier, the animals had to wait for 3 s before acquiring the reward. The elapsed time was measured by an experimenter using a digital stopwatch. After the 3-s wait, the experimenter removed the barrier, allowing the rat to approach and consume the reward. After replacing the barrier and then re-baiting the food cup, the experimenter gently guided the animal to the start arm for the next trial. Once the rat was able to finish 16 trials within 20 min, it underwent the surgical implantation of recording electrodes or guide cannulae.

### Surgery

Under anesthesia with isoflurane (4% induction, 1–3% maintenance), rats were mounted on a stereotaxic instrument (David Kopf Instruments, Tujunga, CA). The skull was exposed and adjusted to place bregma and lambda on the same horizontal plane. In Experiment 1, five rats had six individually drivable tetrodes chronically implanted in the right hemisphere dorsal to the MRF (5.2 mm posterior to bregma, 1.3 mm lateral to the midline, and 5.4 mm ventral to the brain surface). In Experiment 2, another two rats were implanted with a single drivable bundle loaded with six tetrodes in the same area. Each tetrode was made by twisting four 20 μm lacquer-coated tungsten wires (California Fine Wire, Grover Beach, CA) and its tips were plated with gold to a final impedance of 0.2–0.4 MΩ (tested at 1 kHz). For Experiment 3, eight rats received bilateral implantation of guide cannulae (26 gauge; Protech International Inc.) aimed at the MRF (5.2 mm posterior, 1.2 mm lateral, and 5.8 mm ventral to bregma). A 33-gauge dummy cannula was inserted into each guide cannula to prevent clogging.

### Delay discounting and reward discrimination tasks

After a week of recovery, all rats were put back on a food-restricted diet. Three separate experiments were conducted with different groups of animals performing various decision-making tasks on the maze. In Experiment 1, a group of 5 rats implanted with recording tetrodes was trained in a delay discounting task, where they chose between a sooner small (SS) reward and a later large (LL) reward. To assess choice performance as a function of delay to the LL reward, three different lengths of delay (10, 20, and 40 s) prior to the LL reward (0.3 mL) were tested in separate blocks of trials. However, the delay to SS reward (0.05 mL) remained constant at 3 s throughout the experiments. In a daily testing session, the three delays before the LL reward were randomly assigned to different blocks, with only one delay used in a given block. Since the rats did not initially know how long they needed to wait for the LL reward, each block began with 10 forced-choice trials followed by 6 or 8 free-choice trials. During the forced-choice trials, five SS and five LL reward trials were pseudorandomly ordered, and only one goal arm was presented in a given trial after lowering the other goal arm. During the free-choice trials, both goal arms were made available, and their choice preference for the LL reward was measured. A choice was considered made when the entire body (excluding the tail) entered a goal arm. To prevent choice reversal during longer delays, an additional barrier was placed at the entrance of the LL reward arm after the rats entered the goal. The three blocks were separated by an inter-block interval of 5–10 min, during which the rats were placed in a holding area adjacent to the T-maze. The spatial location of SS and LL rewards in the maze was held constant within each rat but was counterbalanced across rats.

In Experiment 2, to further examine whether MRF neural activity was influenced by reward magnitude, the delay to the rewards was kept constant regardless of reward size. Two rats implanted with a tetrode bundle were trained in a reward discrimination task, where they were required to discriminate between two goal arms baited with a small (0.05 mL) and a large (0.3 mL) reward. The testing procedures were identical to the delay discounting task except for two modifications: (1) both small and large rewards were equally delayed by 5 or 10 s, and (2) an opaque guillotine door, connected to a fishing string and controlled remotely by the experimenter, regulated access to the reward cup. This setup allowed the experimenter to open the door from a distance, preventing potential interference with the animals’ behavior. One of the two delays was randomly used in the first block, and the other delay was tested in the second block. The location of small and large rewards was randomly selected each day. Each block consisted of 10 forced-choice (5 small and 5 large rewards) and 10 free-choice trials.

In Experiment 3, a group of eight rats implanted with bilateral cannulae into the MRF was initially trained in the delay discounting task. Once the rats demonstrated similar delay-discounting performance as observed in the electrophysiological experiments, the MRF was manipulated with saline (SAL) or muscimol (MUS) injections on four different days. In each drug testing session, four different delays (3, 10, 20, and 40 s) were imposed prior to the LL reward in separate blocks. The 3-s delay was included to examine whether MRF inactivation altered the ability to discriminate large and small rewards that were equally delayed. To limit the number of drug injections and minimize possible effects of the presentation order of the four delays, each drug was tested on two consecutive days with either ascending (3 to 40 s) or descending sequences (40 to 3 s). Each rat received two consecutive days of either SAL or MUS injections in a within-subject design, with both the order of drug injections and the sequence of delays counterbalanced across rats.

### Intracranial microinjection

MUS (1 μg/μL dissolved in saline) was used to temporarily inactivate the MRF (Jo and Lee, 2010; Jo and Mizumori, 2016). A 33G injection cannula extending 1 mm below the tip of the guide cannula was connected to a 10 μL syringe (Hamilton, Reno, NV) via polyethylene tubing (PE 20). Either 0.3 μL of MUS or SAL was bilaterally infused at a rate of 10 μL/h using a microinfusion pump (KD Scientific, Holliston, MA). The injection cannula was left in place for an additional 3 min to allow diffusion of the drugs from its tip. After the drug injections, rats were carefully observed for any behavioral abnormalities in their home cages for 20 min before being placed on the maze.

### Single-unit recording

Neural activity was monitored prior to each recording session while rats were located in the holding area (Norton et al., 2011; Jo et al., 2013; Redila et al., 2015). Recording tetrodes were connected to a preamplifier and neural data were transferred to a Cheetah data acquisition system (Neuralynx). Unit signals were digitized at 16 kHz, amplified 500–6,000 times, and filtered between 0.6 and 6 kHz. Neuronal spikes were acquired for a 2 ms sampling period whenever a voltage deflection from any tetrode channel exceeded a user-defined threshold. Two LEDs were attached to the preamplifier. The LED signals were captured by a camera mounted on the ceiling at a sampling rate of 30 Hz, and subsequent position data were fed to the acquisition system. Once clearly isolated and stable units were found, a daily recording session started. Thresholds to detect spikes were manually adjusted during recording by visually inspecting incoming signals with monitoring software to adequately capture all units. Acceptable spikes were at least twice the amplitude of the background activity. While the rats were performing the task, three salient events including delay onset, delay termination, and reward were recorded in parallel with neural activity. Specifically, timestamps for delay onset and termination were fed into the data stream when an experimenter operated the stopwatch to measure elapsed time during waiting periods. Reward events were timestamped by ‘lick-detectors’ (custom designed by Neuralynx) when the animals first licked chocolate milk in the food cups. At the end of each session, tetrodes were lowered by 40 μm increments, up to 160 μm per day to find new cells. These recording procedures continued until the tetrodes had traversed the MRF region.

### Histology

After completion of all experiments, the final position of each tetrode was marked by electrolytic lesions (15 μA current for 12 s) while all rats were anesthetized under isoflurane. The animals were then perfused transcardially with physiological saline followed by 10% formalin. Their brains were extracted and stored in a 10% formalin-30% sucrose solution at 4°C for 72 h. The brains were cut into coronal sections (40 μm) on a freezing microtome. The serial sections were stained with cresyl violet. Tetrode tracks and marker lesions were identified using photomicrographs taken under digital microscopy. Only cells verified to be recorded in the MRF were included in the data analysis. Cannula placements were also verified in the same way, except without the use of electrolytic lesions.

### Data analysis

Spikes from multiple single units (signal-to-noise ratio >2:1) were isolated by clustering various spike waveform parameters using Offline Sorter (Plexon, Dallas, TX). Spike sorting was performed using features such as peak, valley, peak-to-valley ratio, and principal component analysis, with comparisons made across simultaneously recorded units from four tetrode wires (Jo et al., 2013; Jo et al., 2018; Pyeon et al., 2024). For some units recorded over multiple sessions, the session with the clearest isolation from background noise and other units was used for analysis. Only units showing stable recording across blocks were included. Further analysis of sorted units and statistics were performed with Matlab software (Mathworks, Natick, MA).

To examine delay-dependent changes in firing, spike rates during the entire delay of each trial were converted to *z*-scores relative to the mean firing within each block of all trials. An MRF cell was classified as delay-excited or delay-inhibited if it passed the following two criteria: (1) its average activity during at least one of the delays was greater or less than a *z*-score of 2 (*p* < 0.023), respectively, and (2) no such increased or decreased activity was observed during the 2.5-s window prior to the delay onset. We examined the activity of these three types of neurons during forced trials and free-choice trials, but there was no statistical difference (Supplementary Figure S1). Therefore, to increase the statistical power in analyzing the activity differences between blocks, we combined the two trial types for analysis.

In addition, reward responses were investigated using peri-event time histograms (PETHs; bin width, 50 ms) that were constructed for the 5-s period around reward encounters. An MRF cell was categorized as reward-responsive if its peak firing rate in the PETH was found within a 400 ms epoch after obtaining the reward (−50 to 350 ms) and the firing rates of the epoch were significantly higher than its mean firing for the block of all trials (Wilcoxon signed-rank test). Spearman’s rank correlation coefficients for individual neuronal responses across three blocks of trials were calculated for each time bin of PETHs to test whether their activity changed linearly as the delay to the LL reward increased. The significance of the correlation was estimated using a permutation test in which firing rates of each bin were randomly shuffled across blocks for 1,000 times. A confidence interval of *p* < 0.99 was calculated from correlation coefficients of the shuffled data.

**Figure 2:**
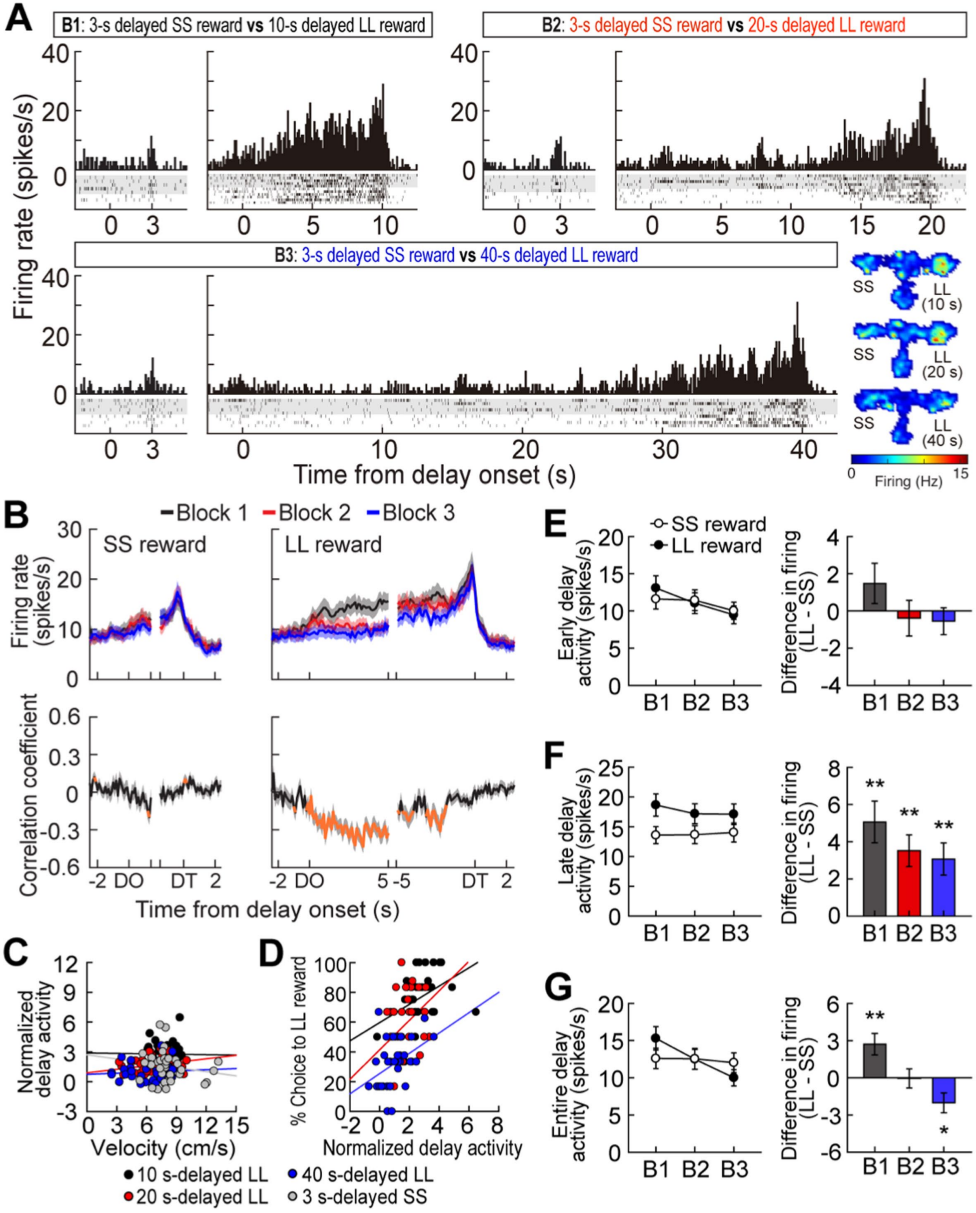
Delay-excited activity in the MRF. **(A)** A representative delay-excited cell. All trials in the histograms (bin width, 100 ms) were aligned to delay onset. Spatial firing rate maps in three blocks of trials showed that the delay-excited cell mainly fired during delays preceding the LL reward. **(B)** Population responses of all delay-excited cells. Data are aligned on delay onset and termination. Correlation coefficients for individual neuronal responses across three blocks were calculated for each time bin. Orange data points that fell outside the 99% confidence interval obtained from a permutation test for at least two consecutive bins were considered significantly correlated. **(C)** Correlations between delay-excited activity and average velocity during delays. Delay-excited activity was normalized to the mean firing rate within each block. **(D)** Correlations between normalized delay-excited activity and choice preference for the LL reward. **(E)** Comparison of the early delay-excited activity measured in the first 1.5 s of delays, using the population histograms in **(B)**. Differential firing rates between LL and SS reward trials within blocks were measured across individual neurons. **(F)** Late delay-excited responses during the last 1.5 s of delays, with differential firing rates between LL and SS reward trials. **(G)** Delay-excited activity during the entire delays, with differential firing rates between LL and SS reward trials. Shaded areas and error bars represent mean ± SEM. ^*^p < 0.05 and ^**^p < 0.01.

**Figure 3:**
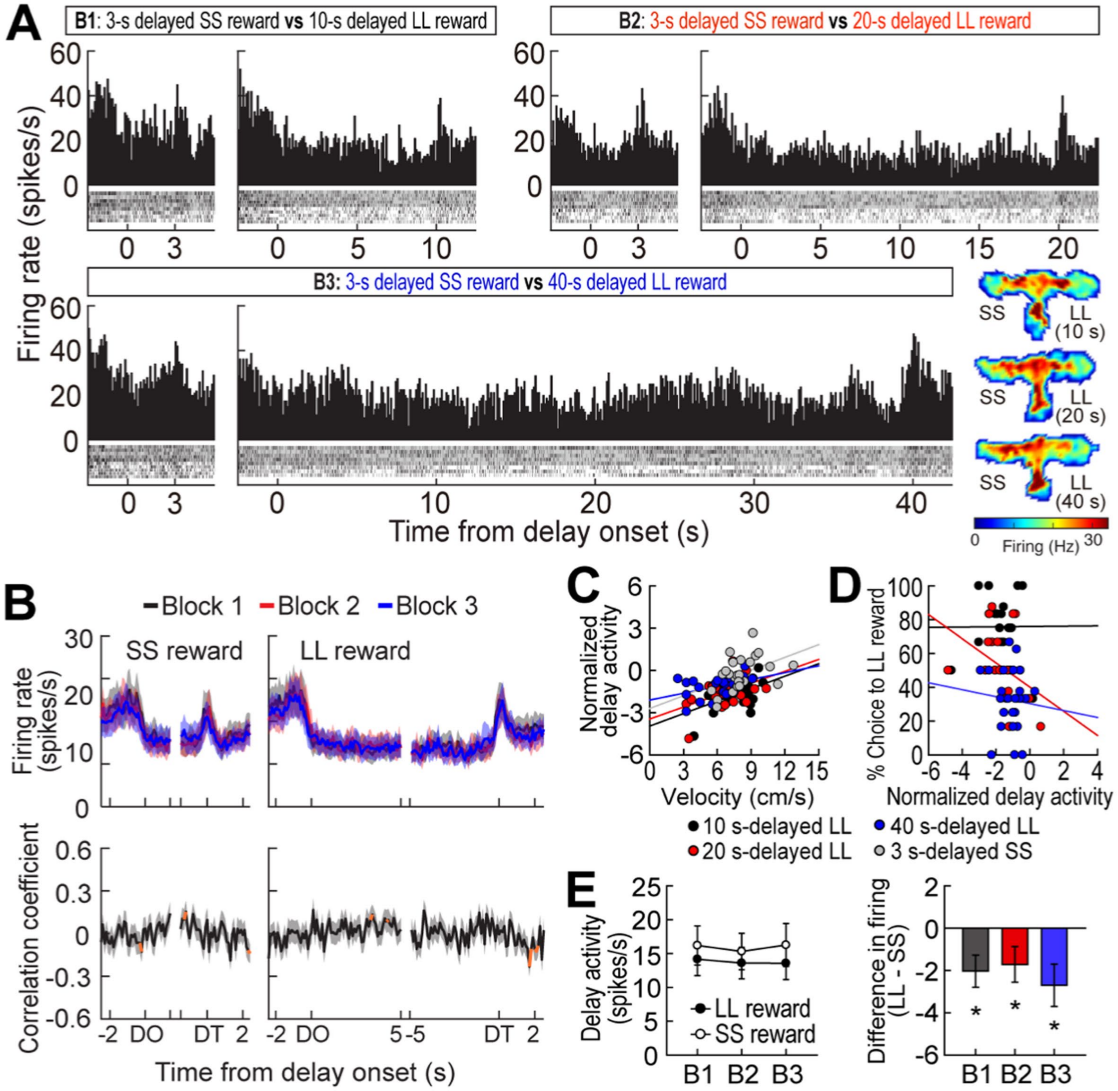
Delay-inhibited activity in the MRF. **(A)** A representative delay-inhibited cell in three blocks of trials. All trials in the histograms (bin width, 100 ms) aligned to delay onset. **(B)** Population responses of all delay-inhibited cells. Data are aligned on delay onset and termination. Significant correlation coefficients for more than two consecutive bins were depicted in orange. **(C)** Correlations between normalized delay-inhibited response and average velocity during delays. **(D)** Correlations between normalized delay-inhibited activity and choice preference for the LL reward. **(E)** Delay-inhibited activity during the entire delays and differential responses between LL and SS reward trials within blocks. Shaded areas and error bars represent mean ± SEM. ^*^p < 0.05.

The rats’ movement during task performance was analyzed by calculating instantaneous velocity, which was determined by the distance between two consecutive head positions sampled at 30 Hz (Pyeon et al., 2023). Spatial firing rate maps of individual cells were also depicted by dividing the number of spikes with the total time spent in each pixel (2.5 cm × 2.5 cm) of position data. Pearson’s correlation tests were conducted to examine the relationship between normalized delay activity and both velocity (Figures 2C, 3C) and choice preference for the LL reward (Figures 2D, 3D). Since behavioral performance was represented by a single measure while multiple delay-excited or delay-inhibited cells could be recorded simultaneously, the average firing rates of delay cells in a given session were used to match the behavioral data.

### Statistical analysis

Differential firing of individual neurons across different reward conditions was analyzed with *t*-tests and repeated-measures ANOVAs followed by Bonferroni’s *post hoc* pairwise comparisons. Spearman’s rank and Pearson’s correlation tests were used to analyze relationships within neuronal activity and between neuronal activity and behavioral performance, respectively. Two-tailed *p*-values <0.05 were considered statistically significant. Data are expressed as mean ± SEM.

## Results

### Experiment 1: choice behavior in the delay discounting task

Five rats were trained to choose between SS and LL rewards on an elevated T-maze (Figure 1A). Three different delays to the LL reward (10, 20, and 40 s) were randomly ordered and tested in separate blocks to examine the animals’ choice performance as a function of delay to the LL reward (Figure 1B). The delay to the SS reward (3 s) remained constant throughout the task. Since an experimenter manually measured the elapsed time of each delay period using a digital stopwatch, there were slight variations in delay length. Across a total of 41 behavioral recording sessions, with each rat undergoing approximately 8 sessions, the SS reward was delayed by 3.19 ± 0.39 s (mean ± SD), and the LL reward was delayed by 10.43 ± 0.49 s, 20.44 ± 0.79 s, or 40.35 ± 0.95 s.

When a 10-s delay was imposed before the LL reward, the animals showed a strong preference for the LL reward (73.9%; Figure 1C). This preference weakened to near chance levels (53.8%) as the LL delay was extended to 20 s. Finally, a 40-s delay to the LL reward reversed choice behavior (32.2%), such that the rats chose the SS reward more often. A Pearson’s correlation test revealed a significant negative correlation between choice performance and delay to the LL reward (*r* = −0.96, *p* < 0.001). In addition to changes in choice behavior, the animals’ physical activity during the delay periods was also monitored. Although the animals engaged with the barrier by sniffing, biting, and rearing against it, they showed minimal movement during the delay periods throughout all sessions (Figure 1D). Overall, the average velocity during the entire delays before the LL reward was lower than that during the entire delays prior to the SS reward (repeated measures ANOVA, *F*_(1,4)_ = 9.51, *p* = 0.037; Figure 1D, inset). There were no differences in velocity across the three delays within the same reward conditions (*F*_(2,8)_ = 1.24, *p* = 0.34) and no interaction between reward size and block (*F*_(2,8)_ = 0.14, *p* = 0.87). These results indicate that the rats exhibited delay discounting, as their preference for the LL reward decreased with increasing delay lengths.

### Experiment 1: delay-excited activity in the delay discounting task

While rats performed the task, a total of 348 cells were recorded from electrodes located in the MRF (Figures 1E,F). Of these cells, 117 cells (33.6%) were significantly excited during at least one of four different lengths of delay relative to their mean firing within the corresponding blocks of trials. A representative delay-excited cell gradually ramped up its firing during delays and reached its peak around the end of the delays (Figure 2A). Then, the ramping activity quickly declined as the barrier was removed. Delay-excited responses were not attributable to delay-dependent changes in behavior, such as decreased velocity during waiting periods, since average velocities during the four different delays were not significantly correlated with average delay-excited firing rates normalized to the mean firing of the corresponding blocks (Pearson’s correlation, absolute *r* values <0.01, *p*-values >0.19; Figure 2C). Instead, higher delay-excited cell activity was correlated with a stronger preference for the LL reward (*r* values >0.39, *p*-values <0.05; Figure 2D).

Delay-excited activity was modulated by delay length (Figure 2B, upper). When Spearman’s rank correlation coefficients for individual neuronal responses across blocks were calculated per time bin of 50 ms, significant negative correlations (Figure 2B, bottom) were found across the three different delays preceding the LL reward, particularly during the first 5 s of the waiting period. However, this pattern was not seen with the SS reward, where the waiting periods remained constant, except for a brief period (200 ms) in the middle of the delays. These results suggest that MRF neurons are involved in reward discounting, with activity reflecting the decreasing value of delayed rewards as wait periods increase. We further analyzed what the delay-excited cells were encoding at specific points during the delay period across all three blocks. During the early phase of the waiting periods (the first 1.5 s after delay onset; Figure 2E), although the reward size (SS or LL) did not have a significant effect (*F*_(1,116)_ = 0.05, *p* = 0.82), there was a significant effect of block (*F*_(2,232)_ = 10.69, *p* < 0.001) and a significant interaction between reward size and block (*F*_(2,232)_ = 3.28, *p* < 0.001). This interaction suggests that delay-excited activity showed different patterns for LL and SS rewards across blocks. While activity for the SS reward remained relatively stable, activity for the LL reward showed a steeper decrease across blocks. This indicates that delay length had a stronger influence on delay-excited activity for the LL reward compared to the SS reward. In contrast, during the late phase of delays (the last 1.5 s before delay termination; Figure 2F), delay-excited cells displayed different levels of peak firing depending on the magnitude of the upcoming rewards. A repeated measures ANOVA confirmed that the final ramping activity prior to the LL reward was significantly higher than that before the SS reward (*F*_(1,116)_ = 24.99, *p* < 0.001). However, the final ramping responses within the same reward conditions were not different, as indicated by no significant effect of block (*F*_(2,232)_ = 0.76, *p* = 0.47) and no interaction between the factors (*F*_(2,232)_ = 2.39, *p* = 0.09).

When average delay-excited activity was measured for the entire delays (Figure 2G), a repeated measures ANOVA found a significant effect of block (*F*_(2,232)_ = 14.28, *p* < 0.001) and a significant interaction (*F*_(2,232)_ = 17.16, *p* < 0.001), without an effect of reward size (*F*_(1,116)_ = 0.1, *p* = 0.75). Interestingly, *post hoc* comparisons revealed that delay-excited activity was significantly greater in the LL reward condition compared to the SS reward condition (*p* = 0.002) when the delay was 10 s, a condition in which rats preferred the LL reward (Figure 1C). When the animals were indifferent between the SS and the 20-s delayed LL reward, there was no significant difference in delay-excited responses between the two reward conditions (*p* = 0.95). Additionally, delay-excited activity was significantly lower in the LL reward condition (*p* = 0.01) when behavioral choices were biased toward the SS reward. Taken together, delay-excited cells in the MRF encode delay length and reward size, with early activity reflecting delay length and late activity correlating with reward size, while activity throughout the entire delay reflects reward discounting.

### Experiment 1: delay-inhibited activity in the delay discounting task

A different group of MRF cells (41/348, 11.8%) was significantly inhibited during at least one of four delays. A representative cell exhibited delay-specific inhibition, followed by short-lasting excitation when the barrier was removed (Figure 3A). As seen from the population activity (Figure 3B), these firing patterns closely resembled the changes in velocity during the waiting periods (Figure 1D). Indeed, normalized delay-inhibited responses were significantly correlated with average velocities in all the four delays prior to both the SS and LL reward (Pearson’s correlation, *r* values >0.42, *p*-values <0.05; Figure 3C). Delay-inhibited cells showed no differential firing across blocks within the same reward conditions, but the average firing rates in the LL reward conditions were lower than those in the SS reward conditions (Figure 3E), as indicated by a significant effect of reward size (*F*_(1,40)_ = 8.79, *p* = 0.005) in a repeated measures ANOVA. However, there was no effect of block (*F*_(2,80)_ = 0.26, *p* = 0.77) and no interaction between the factors (*F*_(2,80)_ = 0.71, *p* = 0.49). The greater inhibition in the LL reward condition was also in line with the significantly lower velocity during waiting periods for the LL reward than for the SS reward (Figure 1D, inset). Furthermore, the delay-inhibited cells had no direct relationship with behavioral performance, since no significant correlations were observed between delay-inhibited responses and choice preference for the LL reward in three blocks of trials (absolute *r* values <0.35, *p*-values >0.09; Figure 3D). Thus, these results indicated that delay-inhibited cells represented the animals’ movement during the task.

### Experiment 1: reward activity in the delay discounting task

It has been reported that MRF cells exhibit phasic responses to reward (Puryear and Mizumori, 2008). In the current study, 39 cells (11.2%) were briefly excited at the time of reward encounters, which is fewer than observed in their previous work. This proportional discrepancy is likely due to differences in the recording sites; the previous report recorded from a ventral area of the MRF, closer to the VTA, while our recordings were taken from a more dorsal region. This ventral location in Puryear’s study likely contributed to the higher number of reward-responsive units observed. Second, it is important to note that the MRF is a highly heterogeneous region involved in a wide array of functions, including motor control, sensory integration, and arousal. Due to this functional diversity, the specific area we recorded from may contain fewer neurons specialized for reward processing. This could explain why we observed a lower proportion of reward-responsive cells.

**Figure 4:**
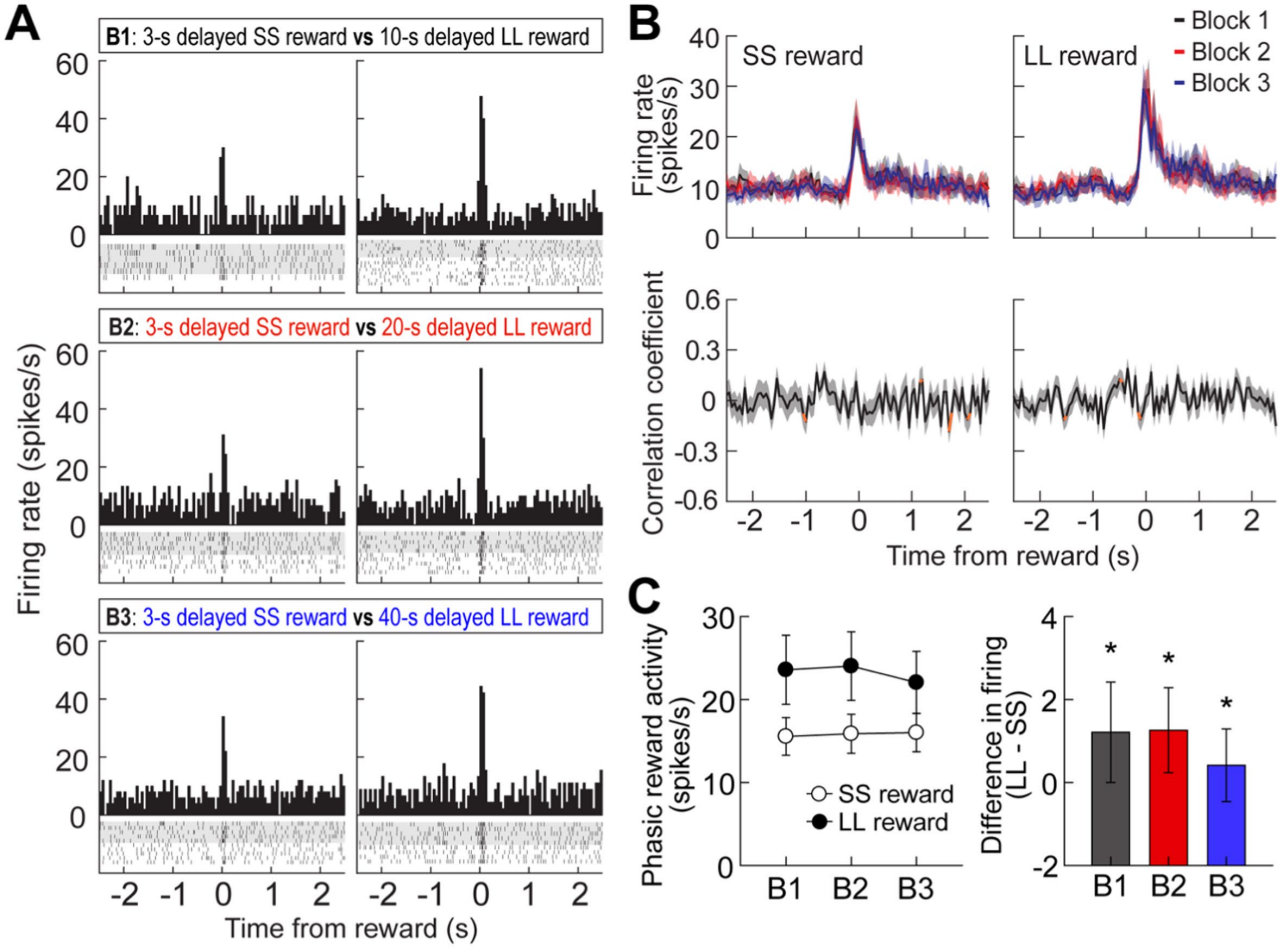
Reward activity in the MRF. **(A)** A representative cell showing reward responses (bin width, 50 ms) in three blocks of trials. **(B)** Population reward responses and correlation coefficients around the time of obtaining rewards. Orange data points indicate significant correlation coefficients **(C)** Average reward activity and differential responses between LL and SS rewards within blocks. Shaded areas and error bars indicate mean ± SEM. ^*^p < 0.05.

As seen from a representative neuron and population responses (Figures 4A,B), reward responses were modulated by the magnitude of rewards regardless of different delays prior to obtaining the rewards. An ANOVA with repeated measures revealed a significant effect of reward size (*F*_(1,38)_ = 9.08, *p* = 0.005; Figure 4C), whereas no effect of block (*F*_(2,76)_ = 0.26, *p* = 0.78) and no interaction between the variables (*F*_(2,76)_ = 1.19, *p* = 0.31) were found. These results suggest that reward-responsive cells encode the absolute value of rewards.

### Experiment 2: responses of MRF cells in the reward discrimination task

**Figure 5:**
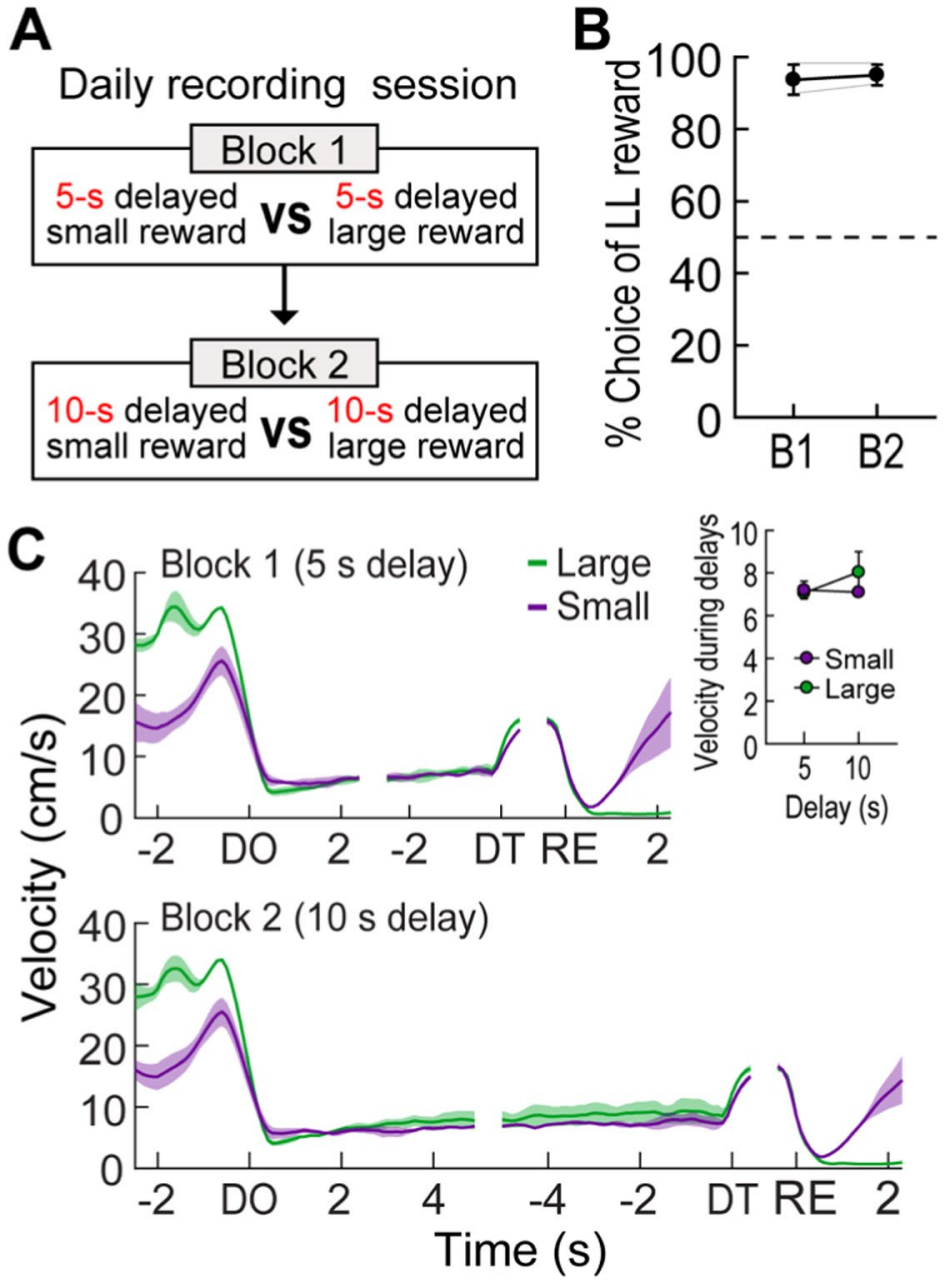
Behavioral performance in a reward discrimination task. **(A)** Daily experimental procedures. The goal arms associated small and large rewards were randomly selected on each day. The rewards were equally delayed, but two different lengths of delay (5 s or 10 s) were randomly used in separate blocks of trials. **(B)** Choice preference for the LL reward in two blocks. **(C)** Velocity traces around waiting periods. Data are aligned on delay onset (DO), delay termination (DT), and reward (RE). The inset plot shows average velocities during the entire waiting periods. Shaded areas and error bars show mean ± SEM.

To further investigate the impact of reward size on the three groups of MRF cells (delay-excited, delay-inhibited, reward-responsive), we held delay times constant while varying reward sizes. Two additional rats, implanted with a single bundle of multiple tetrodes (Figure 1F), were trained in the reward discrimination task. They had to choose between large and small rewards that were equally delayed by 5 or 10 s in separate blocks of trials (Figure 5A). During 33 behavioral recording sessions, the actual delays were 5.17 ± 0.17, and 10.18 ± 0.3 (mean ± SD). The rats showed strong choice biases toward large rewards in both blocks (Figure 5B). The preference for large rewards was also reflected in the animals’ movement. As shown in Figure 5C, they moved faster toward the end of the goal arm associated with large rewards. However, no differences in velocity were observed during waiting periods for both reward sizes, as indicated by no effects of reward size (*F*_(1,1)_ = 0.22, *p* = 0.72) and block (*F*_(1,1)_ = 0.71, *p* = 0.55) as well as no interaction between the factors (*F*_(1,1)_ = 9.04, *p* = 0.2; Figure 5C, inset).

**Figure 6:**
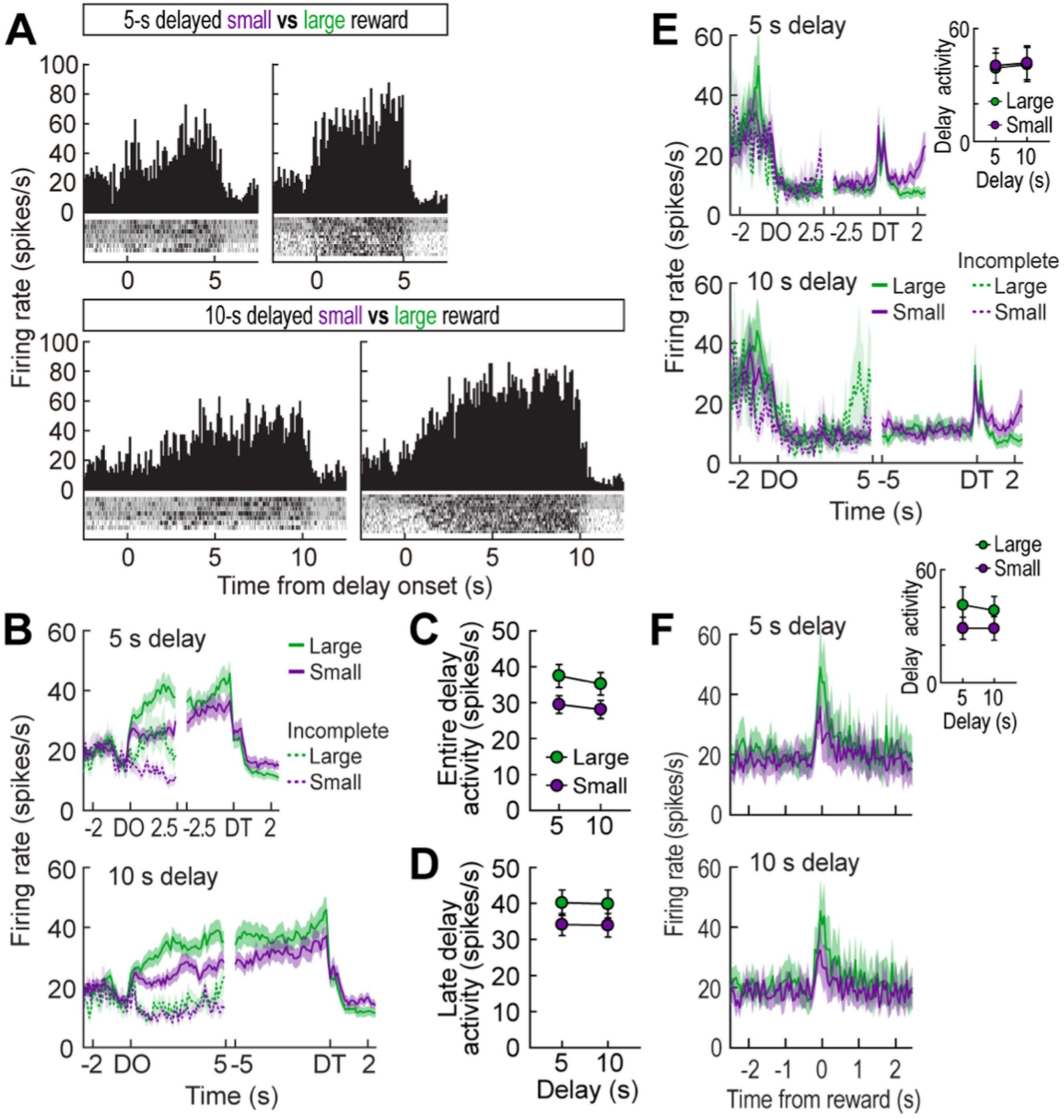
MRF cells in the reward discrimination task. **(A)** A representative delay-excited cell exhibiting ramping activity during delays (bin width, 100 ms). **(B)** Population responses of all delay-excited cells. Histograms are aligned on delay onset (DO) and termination (DT). During the waiting period in the reward discrimination task, there was no additional barrier preventing animals from exiting the arm during the waiting period, so some animals exited early and failed to receive the reward. Neuronal activity in these incomplete trials was marked with a dashed line. **(C)** Average delay-excited responses during the entire delays. **(D)** Late delay-excited activity during the last 1.5 s of delays. **(E)** Population response of all delay-inhibited cells. The inset plot shows average delay-inhibited responses during the entire lengths of delay. **(F)** Population responses of all reward-responsive cells. The inset plot shows average reward responses in two blocks of trials. Shaded areas and error bars show mean ± SEM.

Out of 296 cells recorded in the reward discrimination task, 79 delay-excited cells (26.7%) gradually increased their firing during waiting periods for both large and small rewards, but the magnitude of the ramping activity was strikingly different depending on anticipated reward (Figures 6A,B). A repeated measures ANOVA demonstrated that average delay-excited responses throughout the entire delays were significantly higher in anticipation of larger rewards (*F*_(1,78)_ = 60.92, *p* < 0.001; Figure 6C). A significant effect of block (*F*_(1,78)_ = 7.98, *p* = 0.006) was also found without any interaction between the variables (*F*_(1,78)_ = 1.06, *p* = 0.31), indicating that delay-excited cells were elevated more when the same amounts of reward were expected to be available sooner (i.e., 5 s) rather than later (i.e., 10 s). Interestingly, when delay-excited responses were analyzed during the late phase of delays (the 1.5-s period before the delay termination), delay-excited cells exhibited comparable levels of firing in the same reward conditions irrespective of delay lengths. This observation was confirmed by a significant effect only for reward size (repeated measures ANOVA, *F*_(1,78)_ = 39.99, *p* < 0.001; Figure 6D), but not for block (*F*_(1,78)_ = 0.09, *p* = 0.77). These results suggest that delay-excited cells in the MRF encode the information of expected rewards at the current moment over the course of delays.

In the delay discounting task (Experiment 1), when an animal chose the LL reward, a barrier was manually placed at the entrance of the arm to prevent the animal from exiting the chosen arm. However, in the reward discrimination task (Experiment 2), no barrier was placed to reduce any interference from the experimenter during the trials. As a result, some animals exited the arm before receiving the reward. Therefore, neural activity from trials where animals remained in the arm for at least first half of the delay time was separately analyzed (Figure 6B, dotted line). Among the 79 recorded delay-excited cells, 43 were found in both complete and incomplete trials. When comparing those two trial types, the ramping delay-excited activity was significantly lower in the incomplete trials for both the 5-s delay (significant trial type effect in a repeated measures ANOVA, *F*_(1,84)_ = 27.28, *p* < 0.001) and 10-s delay (*F*_(1,84)_ = 31.14, *p* < 0.001). This absence of ramping activity in incomplete trials suggests that the ramping activity is crucial for anticipation of the upcoming reward and/or maintaining the animals’ motivation to wait throughout the delay period.

Seventeen delay-inhibited (5.7%) and twelve reward-responsive cells (4.1%) were identified in the reward discrimination task. The delay-inhibited cells (Figure 6E) continuously fired in parallel with the animals’ velocity. When the reward was large, the animals moved faster to reach the goal arm (Figure 5C), reflected by increased firing rates in delay-inhibited cells before the delay onset (Figure 6E). However, during the waiting period, both velocity and neural activity decreased similarly in both large and small reward conditions, resulting in no significant group differences. This was supported by a repeated measures ANOVA, which found no significant effects of reward size (*F*_(1,16)_ = 0.26, *p* = 0.62), block (*F*_(1,16)_ = 1.18, *p* = 0.29), or interaction between the factors (*F*_(1,16)_ = 0.03, *p* = 0.86). Reward-responsive cells also consistently signaled different amounts of reward by showing higher phasic responses to larger reward (Figure 6F). Since this group of cells was not influenced by delay length, no distinct changes in reward activity were observed between blocks. A repeated measures ANOVA demonstrated a significant effect of reward size (*F*_(1,11)_ = 9.89, *p* = 0.009), but no effect of block (*F*_(1,11)_ = 0.62, *p* = 0.45) and no interaction between the variables (*F*_(1,11)_ = 0.62, *p* = 0.45). These results corroborated the previous results that delay-inhibited cells represented movement information and reward-responsive cells signaled the value of encountered rewards.

### Experiment 3: effect of MRF inactivation on delay-discounting behavior

**Figure 7:**
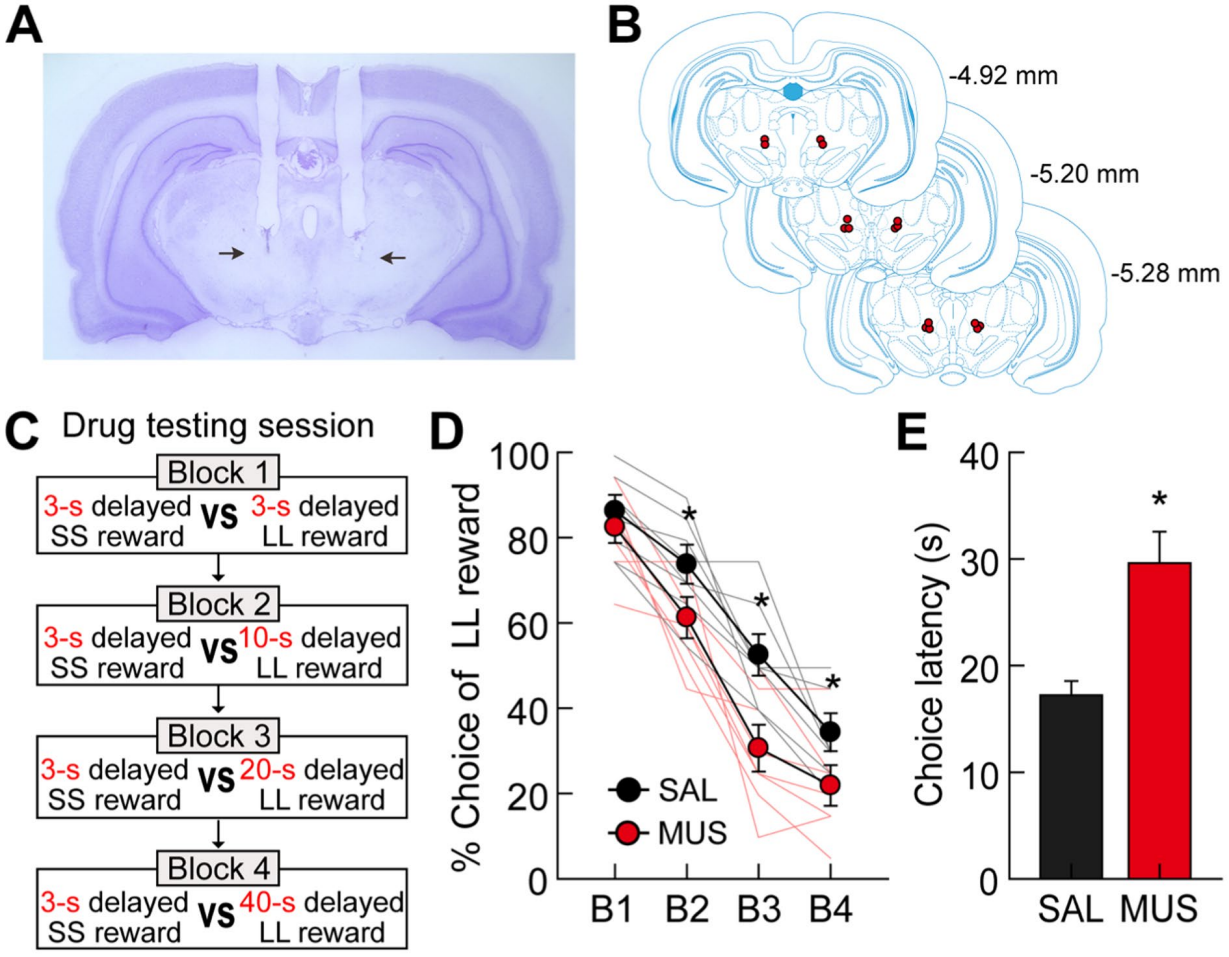
Effects of bilateral inactivation of the MRF on choice performance. **(A)** Nissl-stained section showing the final location of guide cannulae in the MRF (indicated by the black arrows). **(B)** Reconstruction of cannulae implantation with red circle indicating the location. The distances from bregma are indicated on the right side. **(C)** Drug testing procedures. Four different lengths of delay prior to large rewards were used in separate blocks of trials. Each drug was tested with both ascending and descending sequences of the four delays. **(D)** Behavioral performance after either SAL or MUS injection into the MRF. MRF inactivation significantly reduced choice preference for the LL reward. **(E)** Average choice latencies from the start position to the entrance of chosen goal arms. MUS injection into the MRF significantly increased the choice latency compared to SAL injection. All graphs show mean ± SEM.

Because the current recording area contained a proportionally larger number of delay-excited cells, which are thought to play a role during the delay period, we hypothesized that MRF inactivation might influence choice preference for the LL reward by reducing the motivation required to wait through longer delays. To address this hypothesis, 8 rats with bilateral cannulae aimed at the MRF (Figures 7A,B) were tested in a modified version of the delay discounting task, in which a 3-s delay was included in addition to the original three delays to LL reward (Figure 7C). SAL and MUS were used for MRF manipulations (Jo et al., 2007), and each drug was microinfused on two consecutive days to test choice behavior with both ascending and descending sequences of four delays prior to the LL reward. These behavioral data were combined to minimize possible effects of presentation order.

All rats in both drug-injected conditions decreased their preference for the LL reward as the delays to the LL reward increased (Figure 7D). More importantly, MUS injections into the MRF reduced the animals’ choice biases toward the LL reward compared to SAL injections. An ANOVA with repeated measures demonstrated significant effects of block (*F*_(3,21)_ = 140.39, *p* < 0.001) and drug (*F*_(1,7)_ = 25.74, *p* = 0.001), with no interaction between block and drug (*F*_(3,21)_ = 2.35, *p* = 0.1). Moreover, planned comparisons of drug effects within each block (Bonferroni’s *t*-test) found that the preference for the LL reward in MUS-injected conditions was significantly lower across all blocks (*p*-values <0.006) except for one block in which both SS and LL rewards were equally delayed by 3 s (*p* = 0.55). These results indicate that the MRF is critical for delay-based decision making, potentially influencing impulsivity in temporal discounting by reducing the motivation to wait for delayed rewards. However, the MRF does not appear to affect the ability to discriminate locations associated with different reward values.

It should be noted that MRF inactivation also affected other aspects of the animals’ behavior on the maze. Specifically, when average latencies from leaving the start position to entering a chosen goal arm were measured on drug-testing days, MUS infusions significantly increased the times taken for the rats to make choices compared to SAL infusions (paired *t*-test, *t*_(7)_ = 6.16, *p* < 0.001; Figure 7E). We suggest that the increased choice latency may result from alterations in movement-related activity by delay-inhibited cells and/or reduced motivation for delayed rewards following the inactivation of delay-excited cells. However, MUS-injected rats consumed all rewards available in the food cups, indicating no effect of MRF inactivation on consummatory behavior.

## Discussion

The MRF has long been implicated in motor (Fabre et al., 1983), and motivational processes (Olds et al., 1969; Phillips and Olds, 1969; Puryear and Mizumori, 2008). Consistent with this view, three groups of MRF neurons were correlated with such functions in the present decision-making tasks for variously delayed rewards. Delay-inhibited cells represented locomotor activity by tightly firing in parallel with the rats’ velocity on the maze, while reward-responsive cells encoded the magnitude of obtained rewards. In addition, a proportionally larger number of delay-excited cells signaled information about expected rewards during waiting periods. Their ramping responses were initially elevated with different slopes in anticipation of differently delayed rewards. However, their peak firing at the end of the delays reached similar levels when equally sized rewards were expected regardless of the different delay lengths preceding the rewards. Average delay-excited activity during the entire delays signaled the discounted value of expected rewards. Accordingly, when delay-excited cells showed stronger responses during delays to the LL reward, rats tended to choose the reward more often. These firing properties suggest that delay-excited cells encode the motivation of reinforcing future events. In agreement with the electrophysiological results, bilateral inactivation of the MRF altered the animals’ movement on the maze and decreased their choice preference for the LL reward.

It is still possible that delay-excited cells reflected other aspects of behavior, emotion, or cognition that occurred during delays, instead of the current motivational aspect. For instance, the delay-excited activity might reflect an efference copy of the motor command for upcoming approach behavior after the termination of delays (Miles and Evarts, 1979). This would predict identical neural activity during the late phase of delays for both SS and LL rewards, as the rats travel the same distance regardless of reward size once the barriers are removed (Figure 1D). However, the final delay-excited responses varied with reward size, making the efference copy explanation unlikely. Another possibility is that delay-excited cells signal negative arousal, such as gradual buildup of frustration while waiting. However, this cannot account for the positive correlation between delay-excited activity and the preference for the LL reward (Figure 2D). Overall, the firing patterns of delay-excited cells in the MRF are best explained by their role in encoding motivational drive, which integrates information about upcoming reward.

In the reward discrimination task, no barriers were placed to prevent animals from exiting the arm before the delay ended. As a result, some animals exited the arm prematurely and failed to obtain the reward at both the 5-s and 10-s delays. Animals that successfully waited and received the reward displayed sustained ramping activity of delay-excited cells in the MRF throughout the delay period, which reflects the expected value of the chosen outcome and maintains their willingness to wait. In contrast, animals that exited early showed little to no delay-excited cell activity during the waiting period (Figure 6B, dotted lines). This suggests that delay-excited cells encode the motivational aspect of the upcoming reward, which is essential for maintaining motivation to wait until the end of the delay period. Furthermore, inactivation of these neurons increased the time taken to choose the reward (SS or LL), indicating that the animals took longer to make their choice due to decreased motivation caused by the inactivation of MRF neurons (Figure 7E). Similarly, in the delay discounting task, where barriers ensured the animals could not exit early, the MRF’s motivational influence was evident. The most pronounced ramping activity of MRF cells occurred when the animals opted for the LL reward with the shortest delay, indicating their highest preference and strongest motivation for this option (Figure 2A). These results highlight the critical role of delay-excited cells in maintaining motivation during the waiting period and emphasize their importance in delay-based decision-making.

One concern in the delay discounting task was the potential influence of experimenter interference due to manual manipulation of barriers. In this task, barriers were used to prevent premature exits and control access to rewards when the LL reward was chosen, which required experimenters to be physically present near the maze. This setup raised possibility that the observed ramping activity of MRF cells could be attributed to experimenter interaction rather than pure motivational and reward-related activity. To eliminate this potential confound, the reward discrimination task was designed to minimize human interference. Instead of manually controlled barriers, a guillotine door operated remotely via a fishing string was used to block access to rewards. This allowed experimenters to control the barriers from a distance, reducing direct human contact during critical waiting and reward phases. Despite these differences, the ramping activity of MRF cells before reward delivery was consistent across both tasks (Figures 2B, 3B), indicating that this neural activity was not simply a reaction to experimenter presence. The fact that the MRF cells exhibited similar ramping patterns in both the presence and absence of direct experimenter manipulation supports the conclusion that these neurons are genuinely involved in processing motivation of anticipated rewards.

The MRF demonstrates a distinct capability to encode both the absolute size and relative desirability of rewards. This is evident in how the peak firing rates of delay-excited MRF neurons consistently signal the absolute size of upcoming rewards, while their sustained activity throughout the delay reflects the subjective value of those rewards (Figures 2B, 6B). This dual representation of absolute and relative reward values differentiates the MRF from regions like the orbitofrontal cortex (OFC), which primarily focuses on relative reward values (Roesch et al., 2006). Interestingly, our data suggest that the MRF does not directly compare decision values at the moment of choice as spatial firing maps revealed no significant changes in the MRF neuron activity at critical decision-making points, such as the start arm or maze junctions (Figure 2A). This implies that regions like the OFC and VTA may handle the initial value comparisons before transmitting this information to the MRF. The existence of direct anatomical connections between the MRF and both the OFC and VTA further supports this functional interaction (Jones and Yang, 1985; Vertes, 2004; Hoover and Vertes, 2011). Through these connections, the MRF may receive processed information about relative reward values from the OFC and reward prediction signals from the VTA. By integrating these inputs, the MRF effectively processes motivational and reward-related signals, serving as a critical nexus in the neural circuitry of reward-based decision-making.

MRF neurons appear to respond differently depending on the phase of the delay period. Late-phase MRF activity seemed to reflect reward size, with neurons responding in a way that corresponded to the magnitude of the anticipated reward. Interestingly, early-phase activity was notably absent in animals that did not complete the trial in Experiment 2 (Figure 6B), suggesting that early-phase MRF neuron activity may be crucial for sustaining engagement through the delay. This absence of early activity in non-completing animals could be related to impulsivity, as animals lacking sufficient motivation may have been more likely to exit the trial before the reward was delivered. These findings indicate that MRF neurons may play distinct roles at different points in time during the decision-making process. Optogenetic manipulation, which has proven useful in inhibiting neural activity with precise temporal control in other studies (Heymann et al., 2020; Jo et al., 2020), could be particularly valuable for future studies to target specific phases of the delay—early, mid, and late. This approach would provide valuable insights into how MRF neurons contribute to both motivational processes and reward-related decision-making.

In conclusion, the present study demonstrates the involvement of the MRF in delay-based decision-making processes. MRF neurons play a crucial role in sustaining motivation during waiting periods, actively encoding the upcoming reward value and influencing behavior. These findings provide insights into the neural mechanisms supporting delayed gratification and highlight the unique role of the MRF in reward-based decision-making.

## Data Availability

The original contributions presented in the study are included in the article/Supplementary material, further inquiries can be directed to the corresponding authors.

## Supplementary material

SUPPLEMENTARY FIGURE S1 **(A–C)** Population responses of delay-excited cells **(A)**, delay-inhibited cells **(B)**, and reward-responsive cells **(C)**. The upper graphs represent the average activity from forced-choice trials, and the bottom graphs represent the average activity from free-choice trials. **(D–F)** Average firing rates during SS and LL trials for both forced- and free-choice conditions across blocks for delay-excited cells **(D)**, delay-inhibited cells **(E)**, and reward-responsive cells **(F)**. No significant differences were observed between SS and LL trials across blocks (*p*-values >0.05).

## References

[ref1] Baxter M. G. Murray E. A. (2002). The amygdala and reward. Nat. Rev. Neurosci. 3, 563–573. doi: 10.1038/nrn875 12094212

[ref2] Euston D. R. Gruber A. J. McNaughton B. L. (2012). The role of medial prefrontal cortex in memory and decision making. Neuron 76, 1057–1070. doi: 10.1016/j.neuron.2012.12.002, PMID: 23259943 PMC3562704

[ref3] Fabre M. Rolls E. Ashton J. Williams G. (1983). Activity of neurons in the ventral tegmental region of the behaving monkey. Behav. Brain Res. 9, 213–235. doi: 10.1016/0166-4328(83)90129-8, PMID: 6309194

[ref4] Fiorillo C. D. Newsome W. T. Schultz W. (2008). The temporal precision of reward prediction in dopamine neurons. Nat. Neurosci. 11, 966–973. doi: 10.1038/nn.2159, PMID: 18660807

[ref5] Gan J. O. Walton M. E. Phillips P. E. (2010). Dissociable cost and benefit encoding of future rewards by mesolimbic dopamine. Nat. Neurosci. 13, 25–27. doi: 10.1038/nn.2460, PMID: 19904261 PMC2800310

[ref6] Heymann G. Jo Y. S. Reichard K. L. McFarland N. Chavkin C. Palmiter R. D.. (2020). Synergy of distinct dopamine projection populations in behavioral reinforcement. Neuron 105, 909–920.e5. doi: 10.1016/j.neuron.2019.11.024, PMID: 31879163 PMC7060117

[ref7] Hoover W. B. Vertes R. P. (2011). Projections of the medial orbital and ventral orbital cortex in the rat. J. Comp. Neurol. 519, 3766–3801. doi: 10.1002/cne.22733, PMID: 21800317

[ref8] Jo Y. S. Heymann G. Zweifel L. S. (2018). Dopamine neurons reflect the uncertainty in fear generalization. Neuron 100, 916–925.e3. doi: 10.1016/j.neuron.2018.09.028, PMID: 30318411 PMC6226002

[ref9] Jo Y. S. Lee I. (2010). Disconnection of the hippocampal-perirhinal cortical circuits severely disrupts object-place paired associative memory. J. Neurosci. 30, 9850–9858. doi: 10.1523/JNEUROSCI.1580-10.2010, PMID: 20660267 PMC2913067

[ref10] Jo Y. S. Lee J. Mizumori S. J. (2013). Effects of prefrontal cortical inactivation on neural activity in the ventral tegmental area. J. Neurosci. 33, 8159–8171. doi: 10.1523/JNEUROSCI.0118-13.2013, PMID: 23658156 PMC3675177

[ref11] Jo Y. S. Mizumori S. J. (2016). Prefrontal regulation of neuronal activity in the ventral tegmental area. Cereb. Cortex 26, 4057–4068. doi: 10.1093/cercor/bhv215, PMID: 26400913 PMC5028001

[ref12] Jo Y. S. Namboodiri V. M. K. Stuber G. D. Zweifel L. S. (2020). Persistent activation of central amygdala CRF neurons helps drive the immediate fear extinction deficit. Nat. Commun. 11:422. doi: 10.1038/s41467-020-14393-y, PMID: 31969571 PMC6976644

[ref13] Jo Y. S. Park E. H. Kim I. H. Park S. K. Kim H. Kim H. T.. (2007). The medial prefrontal cortex is involved in spatial memory retrieval under partial-cue conditions. J. Neurosci. 27, 13567–13578. doi: 10.1523/JNEUROSCI.3589-07.2007, PMID: 18057214 PMC6673110

[ref14] Jones B. E. Yang T. Z. (1985). The efferent projections from the reticular formation and the locus coeruleus studied by anterograde and retrograde axonal transport in the rat. J. Comp. Neurol. 242, 56–92. doi: 10.1002/cne.902420105 2416786

[ref15] Kinomura S. Larsson J. Gulyas B. Roland P. E. (1996). Activation by attention of the human reticular formation and thalamic intralaminar nuclei. Science 271, 512–515. doi: 10.1126/science.271.5248.512, PMID: 8560267

[ref16] Kobayashi S. Schultz W. (2008). Influence of reward delays on responses of dopamine neurons. J. Neurosci. 28, 7837–7846. doi: 10.1523/JNEUROSCI.1600-08.2008, PMID: 18667616 PMC3844811

[ref17] Miles F. A. Evarts E. V. (1979). Concepts of motor organization. Annu. Rev. Psychol. 30, 327–362. doi: 10.1146/annurev.ps.30.020179.001551 375812

[ref18] Moruzzi G. Magoun H. W. (1949). Brain stem reticular formation and activation of the EEG. Electroencephalogr. Clin. Neurophysiol. 1, 455–473. doi: 10.1016/0013-4694(49)90219-9 18421835

[ref19] Murray E. A. (2007). The amygdala, reward and emotion. Trends Cogn. Sci. 11, 489–497. doi: 10.1016/j.tics.2007.08.013 17988930

[ref20] Narayanan N. Horst N. Laubach M. (2006). Reversible inactivations of rat medial prefrontal cortex impair the ability to wait for a stimulus. Neuroscience 139, 865–876. doi: 10.1016/j.neuroscience.2005.11.072 16500029

[ref21] Norton A. B. Jo Y. S. Clark E. W. Taylor C. A. Mizumori S. J. (2011). Independent neural coding of reward and movement by pedunculopontine tegmental nucleus neurons in freely navigating rats. Eur. J. Neurosci. 33, 1885–1896. doi: 10.1111/j.1460-9568.2011.07649.x 21395868 PMC3095748

[ref22] Olds J. Mink W. D. Best P. J. (1969). Single unit patterns during anticipatory behavior. Electroencephalogr. Clin. Neurophysiol. 26, 144–158. doi: 10.1016/0013-4694(69)90205-3 4183368

[ref23] Peterson B. W. (1984). “The reticulospinal system and its role in the control of movement” in Brainstem control of spinal cord function (Oxford: Elsevier), 27–86.

[ref24] Phillips M. I. Olds J. (1969). Unit activity: motivation-dependent responses from midbrain neurons. Science 165, 1269–1271. doi: 10.1126/science.165.3899.1269, PMID: 5803539

[ref25] Pragay E. B. Mirsky A. F. Ray C. L. Turner D. F. Mirsky C. V. (1978). Neuronal activity in the brain stem reticular formation during performance of a “go-no go” visual attention task in the monkey. Exp. Neurol. 60, 83–95. doi: 10.1016/0014-4886(78)90170-X, PMID: 95962

[ref26] Puryear C. B. Mizumori S. J. (2008). Reward prediction error signals by reticular formation neurons. Learn. Mem. 15, 895–898. doi: 10.1101/lm.1072808, PMID: 19050161 PMC2632841

[ref27] Pyeon G. H. Kim J.-H. Choi J.-S. Jo Y. S. (2024). Activation of CGRP neurons in the parabrachial nucleus suppresses addictive behavior. Proc. Natl. Acad. Sci. U.S.A. 121:e2401929121. doi: 10.1073/pnas.2401929121, PMID: 38843183 PMC11181112

[ref28] Pyeon G. H. Lee J. Jo Y. S. Choi J.-S. (2023). Conditioned flight response in female rats to naturalistic threat is estrous-cycle dependent. Sci. Rep. 13:20988. doi: 10.1038/s41598-023-47591-x, PMID: 38017045 PMC10684534

[ref29] Ray C. L. Mirsky A. F. Pragay E. B. (1982). Functional analysis of attention-related unit activity in the reticular formation of the monkey. Exp. Neurol. 77, 544–562. doi: 10.1016/0014-4886(82)90227-8, PMID: 7117462

[ref30] Redila V. Kinzel C. Jo Y. S. Puryear C. B. Mizumori S. J. (2015). A role for the lateral dorsal tegmentum in memory and decision neural circuitry. Neurobiol. Learn. Mem. 117, 93–108. doi: 10.1016/j.nlm.2014.05.009, PMID: 24910282 PMC5327698

[ref31] Roesch M. R. Calu D. J. Schoenbaum G. (2007). Dopamine neurons encode the better option in rats deciding between differently delayed or sized rewards. Nat. Neurosci. 10, 1615–1624. doi: 10.1038/nn2013, PMID: 18026098 PMC2562672

[ref32] Roesch M. R. Taylor A. R. Schoenbaum G. (2006). Encoding of time-discounted rewards in orbitofrontal cortex is independent of value representation. Neuron 51, 509–520. doi: 10.1016/j.neuron.2006.06.027, PMID: 16908415 PMC2561990

[ref33] Siegel J. M. McGinty D. J. (1977). Pontine reticular formation neurons: relationship of discharge to motor activity. Science 196, 678–680. doi: 10.1126/science.193185, PMID: 193185 PMC9044325

[ref34] Steriade M. McCarley R. W. (1990). Brainstem control of wakefulness and sleep. New York: Plenum Press.

[ref35] Vertes R. P. (2004). Differential projections of the infralimbic and prelimbic cortex in the rat. Synapse 51, 32–58. doi: 10.1002/syn.10279, PMID: 14579424

