# Supplementary figures and images for: A role for the midbrain reticular formation in delay-based decision making
Source: Front Syst Neurosci. 2024 Dec 4;18:1481585. doi: 10.3389/fnsys.2024.1481585 (PMC11652490; doi:10.3389/fnsys.2024.1481585)

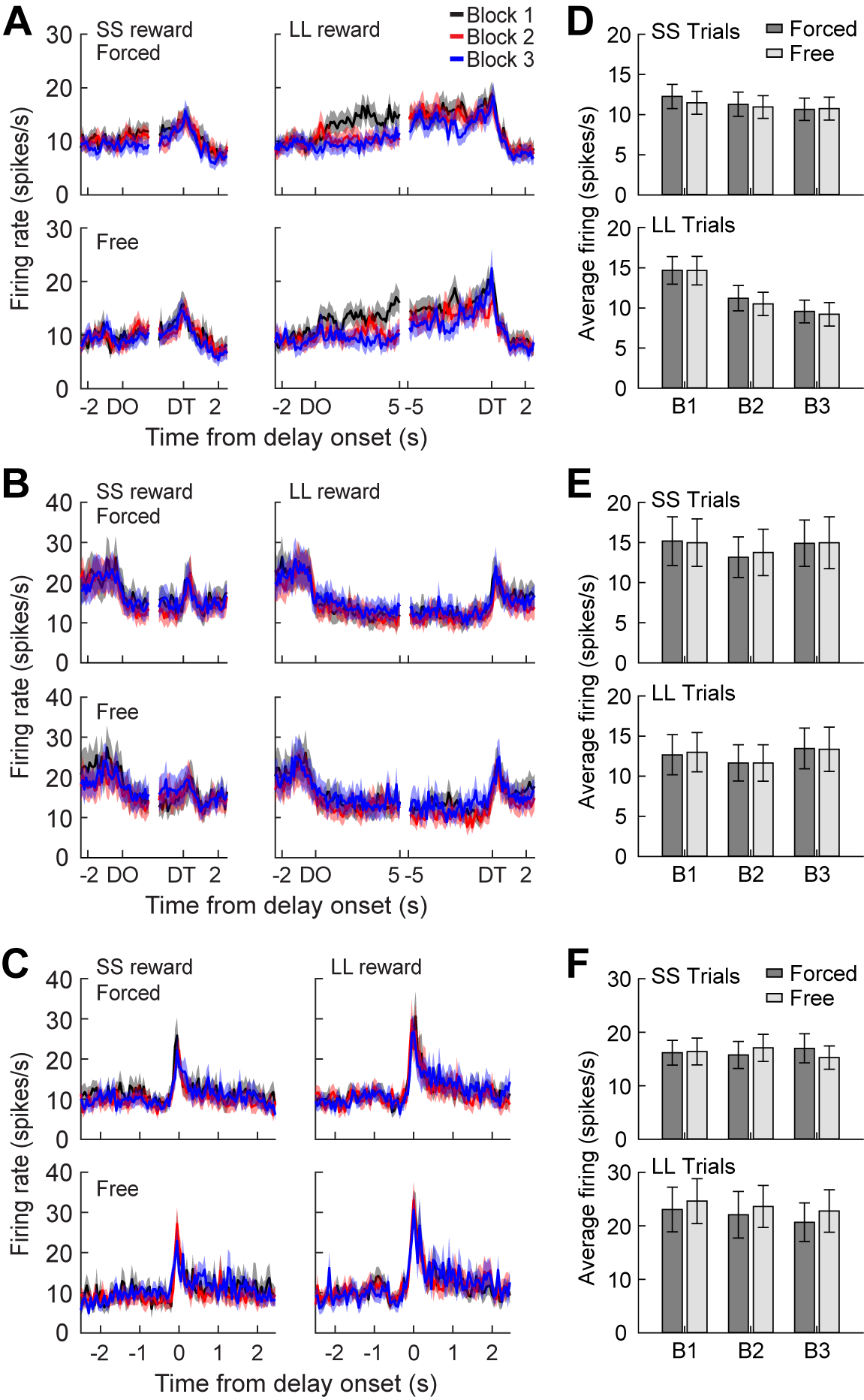

Supplement: SUPPLEMENTARY FIGURE S1 — (A–C) Population responses of delay-excited cells (A), delay-inhibited cells (B), and reward-responsive cells (C). The upper graphs represent the average activity from forced-choice trials, and the bottom graphs represent the average activity from free-choice trials. (D–F) Average firing rates during SS and LL trials for both forced- and free-choice conditions across blocks for delay-excited cells (D), delay-inhibited cells (E), and reward-responsive cells (F). No significant differences were observed between SS and LL trials across blocks (p-values >0.05). [file Image_1.TIF]
